# Radical-Mediated Reactions of α-Bromo Aluminium Thioacetals, α-Bromothioesters, and Xanthates for Thiolactone Synthesis

**DOI:** 10.3390/molecules23040897

**Published:** 2018-04-13

**Authors:** Ruairí O. McCourt, Fabrice Dénès, Eoin M. Scanlan

**Affiliations:** 1 School of Chemistry, Trinity Biomedical Sciences Institute (TBSI), Trinity College Dublin, The University of Dublin, 152-160 Pearse Street, Dublin 2, Ireland; mccourro@tcd.ie; 2 CEISAM UMR CNRS 6230, Université de Nantes, UFR des Sciences et des Techniques, 2 rue de la Houssinière, BP 92208, 44322 Nantes CEDEX 3, France; Fabrice.Denes@univ-nantes.fr

**Keywords:** radical fragmentation, radical cyclisation, thiyl radical

## Abstract

Thiolactones have attracted considerable attention in recent years as bioactive natural products, lead compounds for drug discovery, molecular probes, and reagents for polymerisation. We have investigated radical-mediated C-C bond forming reactions as a strategy for thiolactone synthesis. Cyclisation of an α-bromo aluminium thioacetal was investigated under radical conditions. It was found that at low temperature, a radical fragmentation and rearrangement process occurs. A putative reaction mechanism involving a previously unreported aluminium templated thiol-ene step for the rearrangement process is presented. Cyclisation reactions of α-bromo thioesters and α-xanthate thioesters under radical mediated conditions furnished the desired thiolactones in moderate yields.

## 1. Introduction

**Scheme 1:**
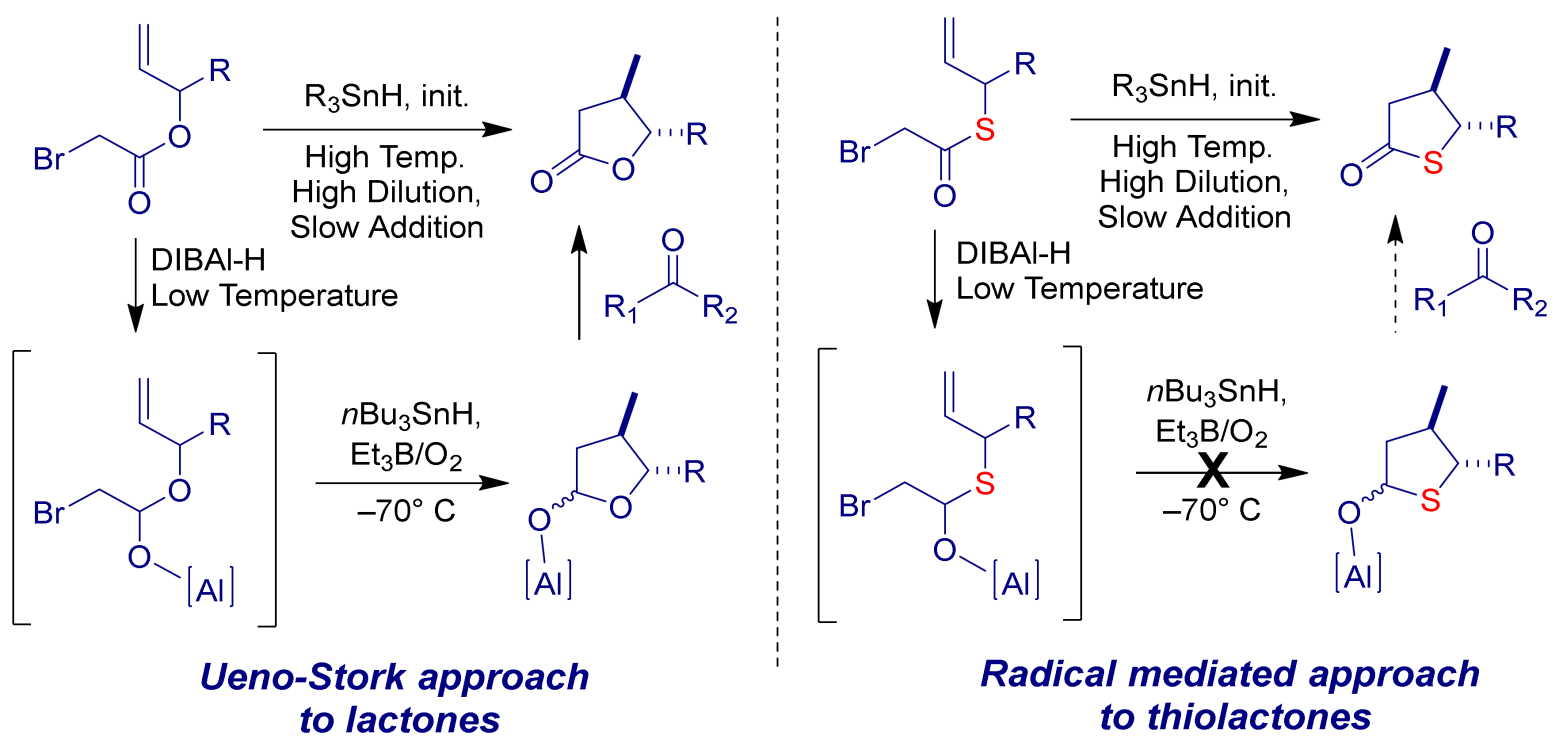
Ueno–Stork approach to lactones and proposed radical-mediated routes to thiolactones.

The 5-*exo-trig* radical cyclisation of allylic α-bromoacetals was reported in 1982 independently by Ueno and Stork [1,2,3]. Since its discovery, this reaction has seen widespread use in organic chemistry, providing a highly efficient method for the synthesis of numerous heterocycles due to its high regioselectivity and diastereoselectivity [3,4]. The cyclic acetal formed through the Ueno-Stork reaction can be readily oxidised to the corresponding lactones using Jones or Grieco’s conditions [5]. The overall process is generally higher yielding than the cyclisation of α-halo-allylic esters and is outlined in Scheme 1. 

Recent work in this field has aimed at improving the overall process, with respect to yield, scope and practicality [6]. It has been established that the α-bromo acetal starting material may be generated in situ from the corresponding esters [7]. It has long been established that at low temperatures (–78 °C), the reduction of esters with diisobutylaluminium hydride (DIBAL-H) furnishes aluminium acetals [8], which decompose upon heating [9]. The reduction of allylic-α-bromo esters to form allylic-α-bromo-aluminium acetals can be coupled with the Ueno–Stork cyclisation to form γ-lactols [7]. This methodology has proven compatible with propargylic α-bromo esters, which undergo 5-*exo*-*dig* cyclisation to form *exo*-alkenes [10]. The methodology was further modified with this reaction being coupled with Oppenauer-type oxidation [11]. After conversion of the allylic (or propargylic) α-bromo aluminium acetal into the lactol-aluminium complex at low temperatures (–78 °C), an excess of a simple sacrificial aldehyde is added, which undergoes Meerwein-Ponndorf-Verley reduction and concomitantly oxidises the aluminium acetal. This one-pot sequence allows for the synthesis of lactones from allylic α-bromo aluminium acetals, often with complete diastereoselectivity [11], as well as polysubstituted butenolides from propargylic α-bromo aluminium acetals [12]. 

We set out to investigate if the α-bromo aluminium acetal methodology developed for lactone synthesis could be applied to allylic α-bromo aluminium thioacetals, in an attempt to synthesise thiolactols suitable for conversion into thiolactones. Thiolactones have emerged as fascinating targets for organic synthesis with intense interest from numerous fields of chemical research, particularly polymer science and medicinal chemistry [13,14,15]. Their synthesis through radical-mediated C–C bond forming reactions has not previously been reported. 

## 2. Results & Discussion

Computational and DOSY experiments support the formation of a dimeric aluminium acetal species for the non-substituted and mono-substituted α-bromo aluminium acetal intermediates involved in the radical cyclisation [16]. Contrary to aluminium acetals derived from esters, the related aluminium thioacetals have proven to be a relatively robust intermediates, demonstrating stability at temperatures up to –15 °C [17]. A dimeric structure was also suggested for the aluminium thioacetal species [17] and it is reasonable to assume that α-halo aluminium thioacetals could be stable enough to serve as precursors in a radical cyclisation. In this case, despite the formation of a beta-sulfanyl radical intermediate susceptible to fragmentation [18,19], the precedent in the literature led us to expect that this process would not compete with the desired cyclisation [20]. 

**Scheme 2:**
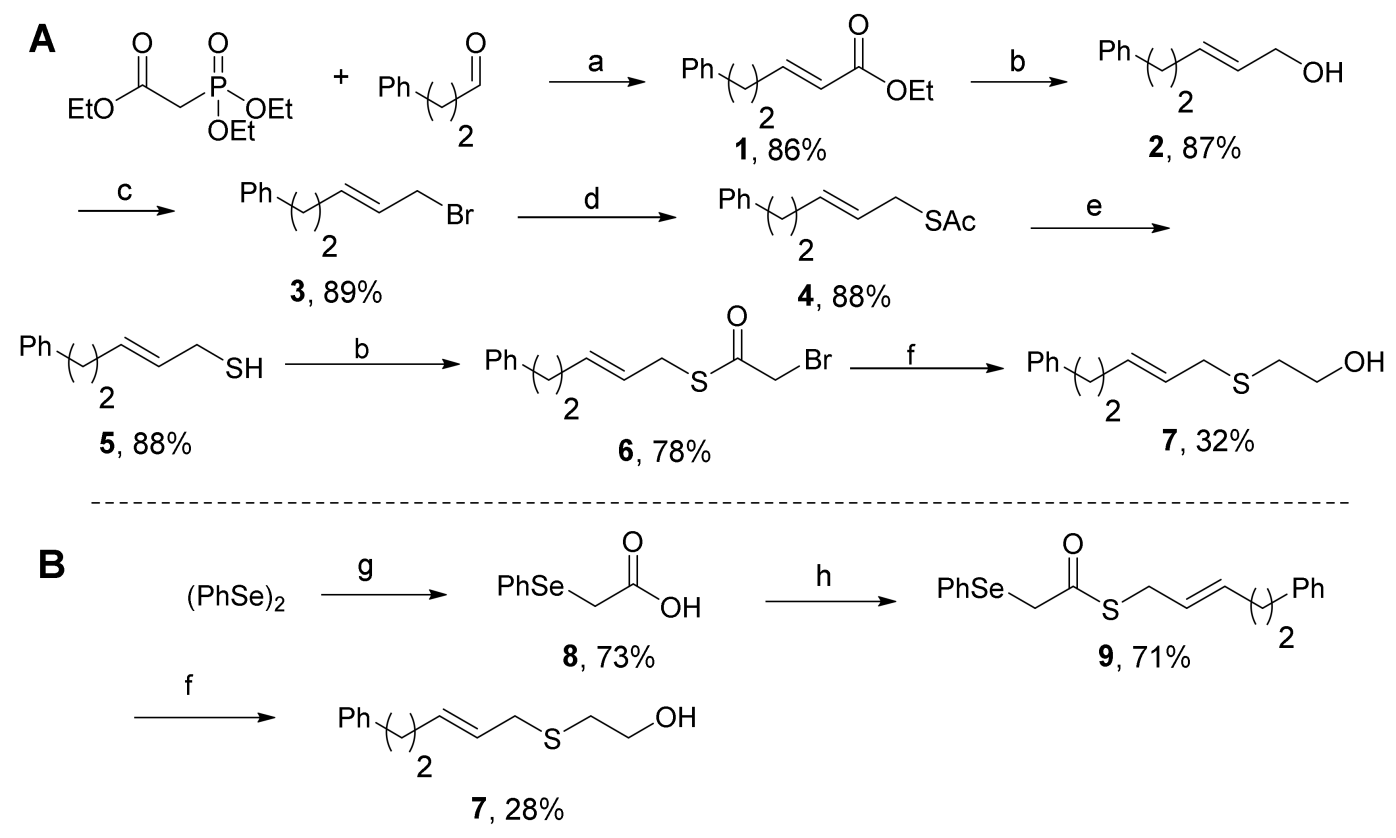
(**A**) The synthesis of an allylic α-bromo-thioester and the attempted Ueno–Stork-type cyclisation. a: *n*BuLi THF, 14 h, 0 °C. b: DIBAl-H, PhMe, −78 °C, 3 h, rt, 15 mins. c: PBr_3_, CH_2_Cl_2_, rt, 3 h. d: KSAc MeCN, rt, 18 h. e: (i) LiAlH_4_, Et_2_O, rt. 2 h. (ii) BrCH_2_CO_2_H, EDC, DMAP, THF, rt, 18 h. f: DIBAL-H, Et_3_B, *n*Bu_3_SnH, air, 14 h. (**B**) The synthesis of an allylic α-phenylselanyl-thioester and its attempted Ueno-Stork cyclisation. g: (i) NaBH_4_, MeOH, 0 °C (ii) BrCH_2_CO_2_H, rt, h: 5, EDC, DMAP, THF, rt, 18 h.

A suitable substrate for investigation of the feasibility of the Ueno-Stork-type cyclisation of α-bromo aluminium thioacetals was prepared starting from hydrocinnamaldehyde in six steps (Scheme 2A). Horner-Wadsworth-Emmons reaction of the corresponding aldehyde gave allyl ester **1** in good yield. Reduction of the ester followed by bromination with PBr_3_ furnished alkyl bromide **3**. Displacement of the bromide with KSAc, followed by reduction of the thioester furnished an allyl thiol, which underwent Steglich coupling with bromoacetic acid to give the desired α-bromo thioester **5** in 78% yield.

When the two-step reduction/cyclisation protocol developed by Dénès et al. was applied to thioester **6**, the thioether **7** was formed in 32% instead of the expected thiolactol. In addition, the allyl thiol **5** was formed in approximately 60%. Repeating the reaction furnished an identical product distribution. Analysis of the reaction mixture following DIBAL-H reduction of **6** at low temperature showed that after this step there was no thioether **7** formed. ^1^H NMR analysis showed only the formation of allyl thiol **5**. The presence of thiol **5** can be explained by the collapse of the aluminium thioacetal generated upon DIBAL-H reduction of thioester **6**. This experiment tends to support the stability of the aluminium thioacetal at low temperature.

In an effort to ascertain the mechanism by which alcohol **7** was formed, α-phenylselanyl carboxylic **8** was prepared from diphenyl diselenide in two steps according to a modified literature procedure [21]. The carboxylic acid was subsequently coupled with the allyl thiol **5** in good yield to give thioester **9** (Scheme 2B).

**Scheme 3:**
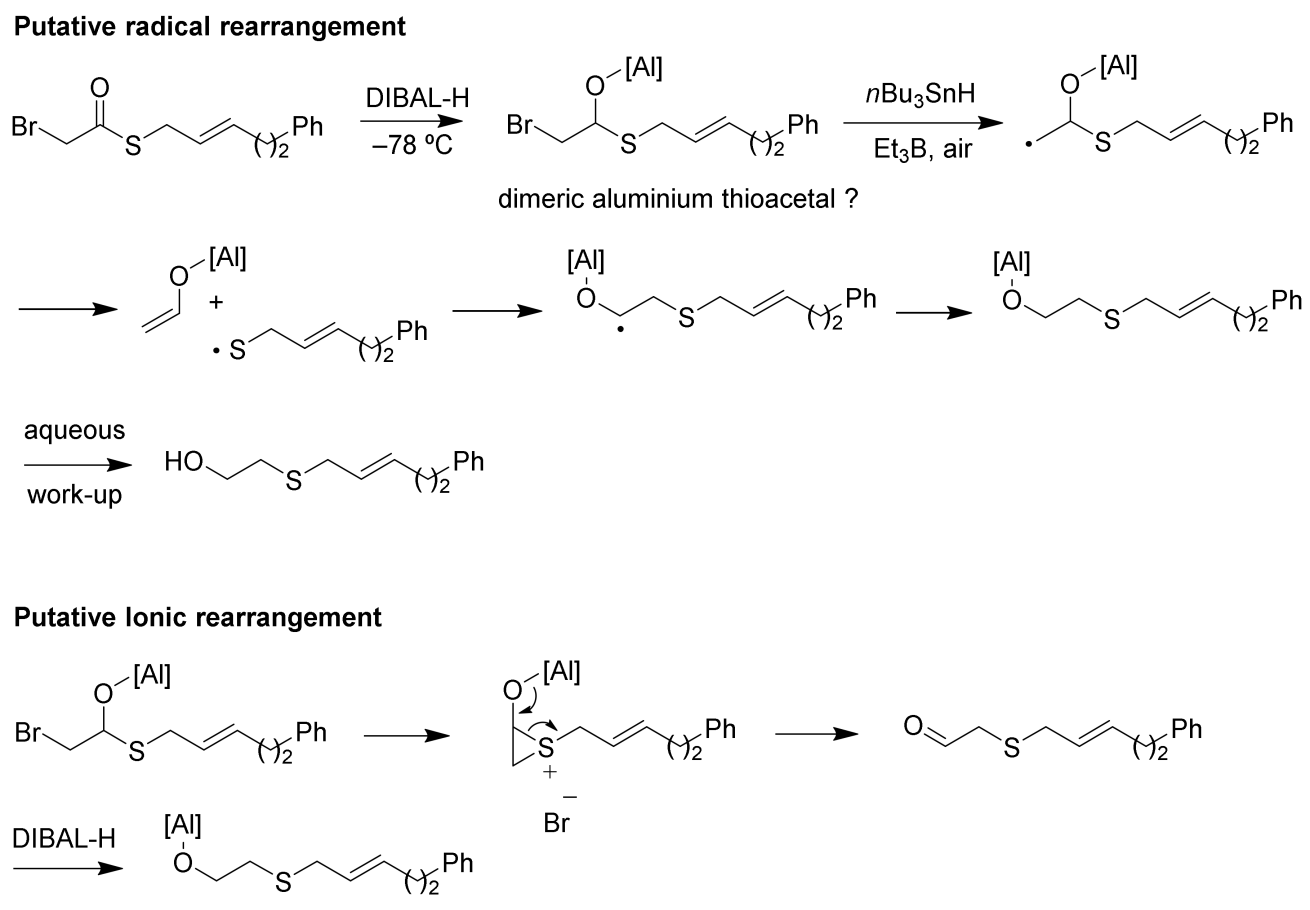
The proposed mechanism leading to rearranged product **7**.

Although the phenylselanyl group reacts in a similar manner to halides under radical conditions (displays similar radical lability), under ionic conditions the reactivities are vastly different. This allowed us to establish by which reaction pathway the formation of **7** was occurring. Under identical conditions, alcohol **6** was formed in 28% yield, providing strong evidence that the transformation occurs via a radical pathway. A proposed mechanism for the rearrangement process for formation of alcohol **7** from thioester **6** is shown in Scheme 3. 

In the first step, DIBAL-H reduction of the thioester to an aluminium thioacetal occurs. Following generation of a tributyltin radical, this intermediate abstracts a bromine atom from the aluminium thioacetal to generate a carbon-centred radical α to the thioacetal (Scheme 2). Contrary to what was observed during the cyclisation of trichloroacetals under atom transfer conditions [20], and despite the fact that the reaction was carried out at low temperature, the aluminium thioacetal subsequently collapses through a homolytic β-fragmentation pathway to generate an aluminium enolate and a thiyl radical, both of which may remain coordinated to the aluminium. The electron-rich alkene thus generated reacts with the electrophilic thiyl radical to give the thiol-ene addition product [22,23]. Hydrogen atom abstraction followed by aqueous work-up furnishes alcohol **7**. The alternative ionic rearrangement involving the nucleophilic displacement of the bromine atom was ruled out by the attempted cyclisation of the more robust seleno-precursor **9**, which led to similar results. No further fragmentation products were observed as collapse of the aluminium intermediate complex, prior to thiol-ene addition would only furnish the observed thiol (following hydrogen atom abstraction) and ethanal, which is reduced by the excess of DIBAL-H to give a volatile by product. The instability of the aluminium complex and the dissociation of the aluminium enolate are the likely factors contributing to the low isolated yield of the radical rearrangement product **7**. However, in light of the results obtained by I. Markó et al. demonstrating the thermal stability of this type of aluminium-thioacetal species, together with the successful cyclisation of protected thioacetals under atom-transfer conditions [20], the fragmentation of the aluminium-thioacetal at the low temperature employed during our reaction is more puzzling. 

**Scheme 4:**
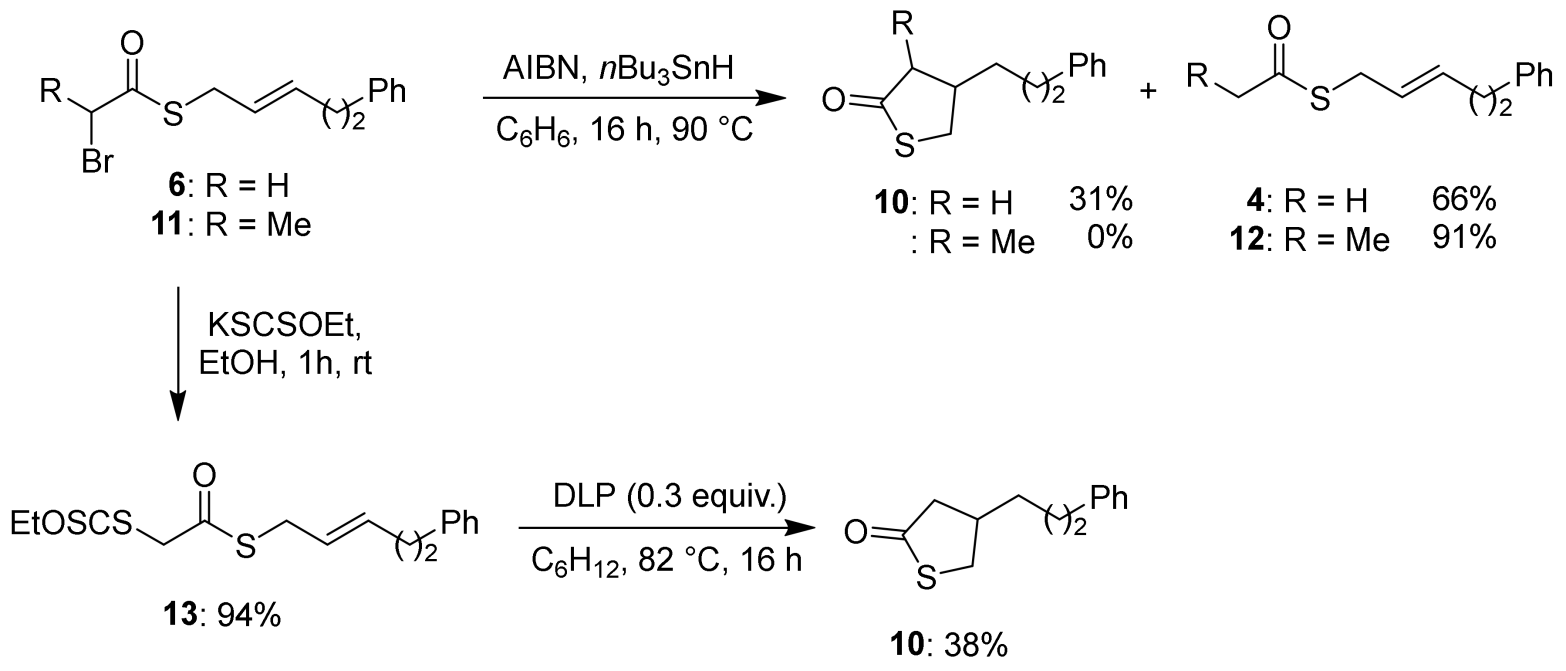
Cyclisation of α-bromo-allylic thioesters under traditional cyclisation conditions.

With the knowledge that the aluminium-thioacetal intermediate was unsuitable for the radical-mediated cyclisation process, we set out to investigate alternative conditions that would avoid this process. The cyclisation of α-bromo esters in the presence of a hydrogen atom donor such as *n*Bu_3_SnH or *n*Bu_3_GeH was investigated by Beckwith in the mid-1980s and it was found that the restricted rotation about the allylic C-O bond in the 2-oxa radical intermediate was responsible for a relatively slow cyclisation [24,25]. As a consequence, dehalogenation prior to the expected radical cyclisation often prevails. Optimized reaction conditions to form the lactones require slow-addition of the *n*Bu_3_SnH in order to maintain the concentration of tin hydride low enough for the intramolecular process to compete favourably [26,27,28]. These optimized reaction conditions were applied to thioester **6** (Scheme 4). We hypothesized that the relatively low rotation barrier in thioesters [29] such as **6** compared to the parent esters and amides [30,31], would facilitate the cyclisation process. Unfortunately, the desired thiolactone **10**, produced via 5-*exo-trig* cyclisation, was obtained in only low yield, with the dehalogenated thioester **4** being afforded as the major product in 66% yield. Attempts to extend this strategy towards the synthesis of other thiolactones failed. For instance, when identical cyclisation conditions were applied to thioester derivative **11**, substituted with a methyl group α to the carbonyl, dehalogenated product **12** was obtained exclusively. This is likely due to the stability of the secondary radical generated, and the *trans-cis* rotation about the carbonyl being more disfavoured in the more substituted **11** allowing for hydrogen atom abstraction to compete with cyclisation. Homolytic β-fragmentation releasing a thiyl radical was not observed in either of these attempted cyclisations, despite the high temperature of the reaction.

Over recent decades, Zard has pioneered and driven the field of radical group transfer of xanthates [32]. These reagents have seen extensive use in intermolecular addition processes as well as in cyclisation reactions [33]. Zard and co-workers have applied this strategy to *N*,*N*-disubstituted α-(xanthyl)-acetamides to prepare lactams [34]. (The preparation of mono- and *bis*-γ-thiolactones by addition of a thiolactone xanthate onto various alkenes has recently been reported) [35]. This approach allows the cyclisation to proceed in the absence of a hydrogen atom donor and thus circumvents the problem associated with the high rotation barrier in amides and esters (For examples of atom transfer reaction for the formation of lactones, see ref [36]). Application of this methodology to the α-xanthate thioester **13**, derived near quantitatively from **6**, was investigated. The radical cyclisation conducted in refluxing cyclohexane in the presence of 0.3 equivalent of dilauroyl peroxide (DLP) as an initiator furnished the reduced lactone **10** with no xanthate being incorporated in the final product. The reduction arises from the hydrogen atom abstraction from the solvent [37]. The use of benzoyl peroxide for the xanthate transfer reaction furnished a complex mixture with no thiolactone product being observed. 

## 3. Materials and Methods

### Instrumental and General Considerations

Unless otherwise stated; all commercial chemicals were obtained from Sigma-Aldrich, Fluka, Fluorochem, Alfa-Aesar, or Fischer Scientific, without further purification. Deuterated solvents for NMR were purchased from Apollo. Dry solvents were distilled under argon and dried over 4 Å molecular sieves prior to use. Solvents for synthesis purposes were used at GPR grade. A Bruker Advance 400 spectrometer, ^1^H (400.13 MHz) and ^13^C (100.6 MHz) and a Bruker Ultrashield 600, ^1^H (600.13 MHz) and ^13^C (150.6 MHz), were employed for NMR spectra. Resonances δ, from the centre point, are in ppm units downfield from an internal reference [38]. NMR data was processed using TopSpin software. Infrared spectra (IR) were recorded on a Perkin Elmer spectrometer. Mass spectrometry analysis was performed with a Q-Tof Premier Waters Maldi-quadrupole time-of-flight (Q-Tof) mass spectrometer equipped with Z-spray electrospray ionisation (ESI) and matrix assisted laser desorption ionisation (MALDI) sources. Silica gel Florisil (200 mesh; Aldrich) was used for column chromatography. Thin-layer chromatography (TLC) was performed using Merck 60 F254 silica gel (pre-coated, 0.2 mm thick, 20 × 20 cm) and visualised by UV light (254 nm), iodine, or molybdenum staining.

*Ethyl (E)-5-phenylpent-2-enoate* (**1**): To a solution of triethyl phosphonoacetate (2.5 mL, 12.5 mmol, 1.25 equiv.) in anhydrous THF (50 mL) under an atmosphere of argon at 0 °C was added 2.5 M *n*BuLi (5.2 mL, 13.0 mmol, 1.3 equiv.) and the mixture was stirred for 30 min. Hydrocinnamaldehyde (1.3 mL, 10 mmol, 1.0 equiv.) was then added, and after 10 mins the reaction mixture was allowed to warm to rt, and was stirred for a further 14 h. The reaction was diluted CH_2_Cl_2_ (250 mL) and quenched with 2 M aqueous HCl (40 mL). After phase separation, the aqueous phase was extracted further with CH_2_Cl_2_ (2 × 50 mL). The combined organic layers were then washed with brine, dried over MgSO_4_, filtered and the solvent removed in vacuo. Purification was achieved by column chromatography on silica gel using 3.0→7.0% EtOAc/Hexane (*v*/*v*) to afford the title compound as yellow wax (1.758 g, 8.62 mmol, 86%). **δ_H_** (400 MHz, CDCl_3_): 7.33–7.25 (m, 2H, Ar-H), 7.24–7.16 (m, 3H, Ar-H), 7.06–6.96 (m, 1H, =CH), 5.85 (d, *J* = 15.6 Hz, 2H, =CH), 4.19 (q, *J* = 7.2 Hz, 2H, -CH_2_CH_3_), 2.78 (t, *J* = 7.5 Hz, 2H, -CH_2_Ph), 2.53 (app-q, 2H, -CH_2_CH=), 1.29 (t, *J* = 7.2 Hz, 2H, CH_3_). **LRMS**: (ESI^−^) *m*/*z* calcd for C_13_H_16_O_2_ ([M + Cl]^−^): 239.1. Found: 239.1. The spectral data were in accordance with those reported in the literature [39].

*(E)-5-phenylpent-2-en-1-ol* (**2**): To a solution of **1** (1.02 g, 5.0 mmol, 1.0 equiv.) in anhydrous CH_2_Cl_2_ (40 mL) under an atmosphere of argon at −78 °C was added 1 M DIBAL-H in CH_2_Cl_2_ (12.5 mL, 12.5 mmol 2.5 equiv.), and the reaction was stirred at this temperature for 3 h. The reaction was then warmed to rt and H_2_O (2 mL), then aqueous 1 M NaOH solution (15 mL), followed again by H_2_O (5 mL), were added, with 5 minutes between each addition, maintaining vigorous stirring. The mixture was then poured into a separating funnel, and after phase separation, the aqueous phase was extracted with CH_2_Cl_2_ (2 × 25 mL). The combined organic layers were then washed with brine, dried over MgSO_4_, filtered through a plug of silica gel, and the solvent removed in vacuo to yield the title compound (705 mg, 4.35 mmol, 87%) without the requirement for further purification. **δ_H_** (400 MHz, CDCl_3_): 7.32–7.25 (m, 2H, Ar-H), 7.22–7.15 (m, 3H, Ar-H), 5.80–5.63 (m, 2H, 2(-CH=)), 4.09 (app-t, 2H, CH_2_OH), 2.72 (t, *J* = 7.7 Hz, 2H, -CH_2_Ph), 2.38 (app-q, 2H, -CH_2_CH=). **LRMS**: (ESI^−^) *m*/*z* calcd for C_11_H_14_ONa ([M + Na]^−^): 185.1. Found: 185.1. The spectral data were in accordance with those reported in the literature [40]. 

*(E)-(5-Bromopent-3-en-1-yl)benzene* (**3**): To a solution of **2** (600 mg, 3.7 mmol, 1.0 equiv.) in anhydrous Et_2_O (10 mL), under an atmosphere of argon at 0 °C was slowly added PBr_3_ (0.22 mL, 2.3 mmol, 0.6 equiv.). The mixture was stirred for 30 mins at this temperature, then poured into a separating funnel, diluted with Et_2_O (25 mL), and quenched with brine. After phase separation, the aqueous phase was extracted with Et_2_O (2 × 25 mL). The combined organic phases were dried over MgSO_4_, filtered, and the solvent removed in vacuo. Purification of the crude product was achieved by column chromatography on silica gel using 5% Et_2_O/Hexane (*v*/*v*) to give the title compound as a colourless oil (738 mg, 3.29 mmol, 89%). **δ_H_** (400 MHz, CDCl_3_): 7.34–7.24 (m, 2H, Ar-H), 7.23–7.13 (m, 3H, Ar-H), 5.94–5.58 (m, 2H, 2(-CH=)), 3.94 (d, 2H, CH_2_Br), 2.71 (t, *J* = 7.6 Hz, 2H, -CH_2_Ph), 2.40 (app-q, 2H, -CH_2_CH=). **LRMS**: (ESI^+^) *m*/*z* calcd for C_11_H_13_BrNa ([M + Na]^+^): 247.0. Found: 247.0. The spectral data were in accordance with those reported in the literature [40]. 

*(E)-S-(5-phenylpent-2-en-1-yl) ethanethioate* (**4**): To a stirred solution of **3** (784 mg, 3.5 mmol, 1.0 equiv,) in DMF (2.5 mL) at room temperature was added KSAc (1.197 g, 10.5 mmol, 3.0 equiv.), and the suspension was stirred vigorously for 18 h. The mixture was diluted with Et_2_O (25 mL), poured into a separating funnel, washed with water (20 mL), then with a 1 M HCl/brine mixture (7 × 50 mL). The organic phase was dried over MgSO_4_, filtered, and the solvent evaporated in vacuo to yield the title compound as a colourless oil (678 mg, 3.08 mmol, 88%) without the requirement for further purification. **δ_H_** (400 MHz, CDCl_3_): 7.31–7.25 (m, 2H, Ar-H), 7.22–7.15 (m, 3H, Ar-H), 5.75–5.65 (m, 1H, -CH=), 5.50–5.40 (m, 1H, -CH=), 3.49 (d, *J* = 7.1 Hz, 2H, -CH_2_SAc), 2.67 (t, *J* = 7.8 Hz, 2H, -CH_2_Ph), 2.37–2.29 (m, 5H, SAc, -CH_2_CH=). **LRMS**: (ESI^+^) *m*/*z* calcd for C_13_H_16_OSNa ([M + Na]^+^): 243.1. Found: 243.1. The spectral data were in accordance with those reported in the literature [41]. 

*(E)-5-phenylpent-2-ene-1-thiol* (**5**): To a solution of **4** (350 mg, 1.59 mmol, 1.0 equiv.) in anhydrous CH_2_Cl_2_ (15 mL) under an atmosphere of argon was added 1 M DIBAL-H (4 mL, 3.9 mmol, 2.5 equiv.) and the mixture was stirred for 2 h at −78 °C. The reaction was then warmed to rt and H_2_O (2 mL), then aqueous 1 M NaOH solution (15 mL), followed again by H_2_O (5 mL), were added, with 5 minutes between each addition, maintaining vigorous stirring. The mixture was then poured into a separating funnel, and after phase separation, the aqueous phase was extracted with CH_2_Cl_2_ (2 × 25 mL). The combined organic layers were then washed with brine, dried over MgSO_4_, filtered through a plug of silica gel, and the solvent removed in vacuo to yield the thiol as a colourless wax (263 mg,1.479 mmol, 93%) without the requirement for further purification. **δ_H_** (400 MHz, CDCl_3_): 7.35–7.28 (m, 2H, Ar-H), 7.25–7.17 (m, 3H, Ar-H), 5.64–5.59 (m, 2H, 2(=CH-)), 3.17–3.11 (m, 2H, -CH_2_SH), 2.72 (t, *J* = 7.8 Hz, 2H, -CH_2_Ph), 2.40–2.33 (m, 2H, -CH_2_CH=), 1.40 (t, *J* = 7.5 Hz, 1H, SH). **δ_C_** (100 MHz, CDCl_3_): 141.7 (q, Ar-C), 131.1 (=CH), 129.8, 128.5, 128.3 (Ar-C), 125.9 (=CH), 35.6 (-CH_2_Ph), 33.9 (-CH_2_CH=), 26.8 (-CH_2_SH). **LRMS**: (ESI^−^): calcd. C_11_H_14_SNa ([M + Cl]^−^): 213.1. Found 213.1.

*(E)-S-(5-Phenylpent-2-en-1-yl)-2-bromoethanethioate* (**6**): To DIC (460 µL, 2.96 mmol, 2.0 equiv.) in anhydrous THF (5.5 mL), under argon was added BrAcOH (411 mg, 2.96 mmol, 2.0 equiv.). The mixture was stirred for 15 min and then **5** (263 mg, 1.48 mmol, 1.0 equiv.) and DMAP (90 mg, 0.74 mmol, 0.5 equiv.) in anhydrous THF (5.5 mL) under an atmosphere of argon were added. The mixture was stirred at rt for 14 h, then diluted with Et_2_O (150 mL), filtered through a plug of silica and the solvent removed in vacuo. The crude product was purified by column chromatography on silica gel using 3% Et_2_O/Hexane (*v*/*v*) to furnish **6** (344 mg, 1.15 mmol, 78%) as a colourless oil. **R_f_** = 0.43 (5% E_2_tO/Hexane). **δ_H_** (400 MHz, CDCl_3_): 7.23–7.07 (m, 5H, Ar-H), 5.83–5.73 (m, 1H, -CH=), 5.53–5.43 (m, 1H, -CH=), 4.20 (s, 2H, -CH_2_Br), 3.59 (d, *J* = 7.4 Hz, 2H, -CH_2_S), 2.71 (t, *J* = 7.9 Hz, 2H, -CH_2_Ph), 2.38 (app-q, 2H, -CH_2_CH=). **δ_C_** (100 MHz, CDCl_3_): 193.6 (C=O), 141.5 (q, Ar-C), 134.6 (=CH-), 128.5, 128.3, 125.9 (Ar-C), 124.3 (=CH-), 48.0 (-CH_2_Br), 35.5 (-CH_2_Ph), 34.0 (-CH_2_S) 31.8 (-CH_2_CH=). **ν_max_** (ATR)/cm^−1^: 608 (C-Br), 1084 (C-S), 1621 (C=C), 1687 (C=O). *m*/*z* **HRMS** (ESI^+^) calcd for C_13_H_19_NOSBr = 316.0365 [M + NH_4_]^+^. Found 316.0364. 

*(E)-2-((5-phenylpent-2-en-1-yl)thio)ethan-1-ol* (**7**): To a stirred solution of **6** (200 mg, 0.66 mmol, 1.0 equiv.) in anhydrous PhMe (10 mL) under argon at −78 °C was added 1 M DIBAL-H in PhMe dropwise (1.0 mL, 1.00 mmol, 1.5 equiv.). After complete disappearance of the starting material was observed by TLC (30 min), 1 M in hexanes Et_3_B (0.33 mL, 0.33 mmol, 0.5 equiv.), *n*Bu_3_SnH (0.27 mL, 1.00 mmol, 1.5 equiv.), and air (*ca* 1 mL) were simultaneously added at −78 °C. The mixture was maintained at this temperature for 14 h. The reaction mixture was then warmed to rt and quenched with saturated NaF solution (30 mL). The mixture was stirred vigorously for 2 h, poured into a separating funnel and diluted with CH_2_Cl_2_ (100 mL). After phase separation, without agitation, the aqueous phase was extracted with CH_2_Cl_2_ (2 × 50 mL). The combined organic layers were then washed with brine (25 mL), dried over MgSO_4_, filtered and the solvent removed in vacuo. The crude product was purified by column chromatography on anhydrous K_2_CO_3_/silica gel (10% (*v*/*v*)) using 35% Et_2_O/Hexane (*v*/*v*) to give **7** (47 mg, 0.21 mmol, 32%) as a colourless oil. **R_f_** = 0.17 (30% Et_2_O/Hexane (*v*/*v*)). **δ_H_** (300 MHz, CDCl_3_): 7.34–7.28 (m, 2H, Ar-H), 7.25–7.17 (m, 3H, Ar-H), 5.64–5.39 (m, 2H, 2 (=CH)), 3.67 (t, *J* = 6.1 Hz, 2H, -CH_2_OH), 3.11 (d, *J* = 6.9 Hz, 2H, -SCH_2_CH=), 2.74 (t, *J* = 7.2 Hz, 2H, -CH_2_Ph), 2.60 (t, *J* = 6.1 Hz, 2H, -CH_2_S-), 2.41 (app-q, 2H, -CH_2_CH=), 2.24 (bs, 1H, OH). **δ_C_** (75 MHz, CDCl_3_): 141.5 (q, Ar-C), 133.2 (=CH-), 128.5, 128.4, 126.5 (Ar-C), 125.9 (=CH-), 60.2 (-CH_2_OH), 35.7 (-CH_2_S-), 33.9 (-CH_2_CH=), 33.5 (-SCH_2_CH=), 33.3 (-CH_2_Ph). ***v*_max_** (ATR)/cm^−1^: 1021 (C-S), 1048 (C-O), 3352 (O-H). *m*/*z* **HRMS** (ESI^+^) calcd for C_13_H_18_OS = 222.1078 (M)^+^. Found 222.1074.

*2-(Phenylselanyl)acetic acid* (**8**): To a stirred solution of (PhSe)_2_ (3.48 g, 11.2 mmol, 1.0 equiv.) in MeOH (40 mL) at 0 °C was added NaBH_4_ (2.00 g, 50.0 mmol, 2.5 equiv.) slowly over 30 mins. After the final addition the reaction was stirred for a further 30 mins. BrAcOH (3.06 g, 11.2 mmol, 1.0 equiv.) in MeOH (8 mL) was then added. The reaction was warmed to rt and stirred for 15 mins. The solvent was removed *in vacuo* and the crude yellow residue was redissolved in Et_2_O (25 mL) and poured in a separating funnel. The organic phase was extracted with water (50 mL). The aqueous phase was then acidified with 4 M HCl and the aquesous phase was extracted with CH_2_Cl_2_ (5 × 20 mL). The combined organic phases were dried over MgSO_4_, filtered, and the solvent removed in vacuo to yield **8** as a yellow oil (1.766 g, 8.18 mmol, 73%) without further purification being required. **δ_H_** (400 MHz, CDCl_3_): 11.3 (bs, 1H, CO_2_H), 7.56–7.50 (m, 2H, Ar-H), 7.26–7.20 (m, 3H, Ar-H), 3.45 (s, 2H, -CH_2_−). **LRMS**: (ESI^−^) *m*/*z* calcd for C_8_H_7_O_2_Se ([M − H]^−^): 215.0. Found: 215.0. The spectral data were in accordance with those reported in the literature [21]. 

*(E)-S-(5-Phenylpent-2-en-1-yl) 2-(phenylselanyl)ethanethioate* (**9**): To a solution of **8** (400 mg, 2.0 mmol, 2.0 equiv.) in anhydrous THF (5.5 mL), under an atmosphere of argon was added DIC (0.6 mL, 4.0 mmol, 2.0 equiv.). The mixture was stirred for 15 min and then **6** (178 mg, 1.0 mmol, 1.0 equiv.) and DMAP (61 mg, 0.5 mmol, 0.5 equiv.) in anhydrous THF (5.5 mL) under an atmosphere of argon were added. The mixture was stirred at rt for 14 h, then diluted with Et_2_O (150 mL), filtered through a plug of silica and the solvent removed in vacuo. The crude product was purified by column chromatography on silica gel using 5% EtOAc/Hexane (*v*/*v*) to furnish **9** (267 mg, 0.71 mmol, 71%) as a yellow oil. **R_f_** = 0.27 (5% Et_2_O/Hexane). **δ_H_** (400 MHz, CDCl_3_): 7.60–7.55 (m, 2H, Ar-H), 7.32–7.25 (m, 5H, Ar-H), 7.22–7.13 (m, 3H, Ar-H), 5.75–5.64 (m, 1H, =CH-), 5.47–5.38 (m, 1H, =CH-), 3.76 (s, 2H, -CH_2_Se), 3.50 (d, *J* = 7.3 Hz, -CH_2_S), 2.67 (t, *J* = 7.8 Hz, 2H, -CH_2_Ph), 2.32 (app-q, 2H, -CH_2_CH=). **δ_C_** (100 MHz, CDCl_3_): 195.4 (C=O), 141.6 (q, Ar-C), 133.9 (=CH-), 133.6, 129.3, 128.5, 128.4 (Ar-C), 128.0 (q, Ar-C), 125.9 (Ar-C), 124.8 (=CH-), 36.5 (-CH_2_Se), 35.5 (-CH_2_Ph), 34.1 (-CH_2_CH=), 32.0 (-CH_2_S). **ν_max_** (ATR)/cm^−1^: 1679 (C=O). *m*/*z* **HRMS** (ESI+) calcd for C_19_H_21_OSSe = 377.0473 [(M + H)]^+^. Found 377.0469.

*4-(3-Phenylpropyl)dihydrothiophen-2(3H)-one* (**10**): To a stirred solution of **6** (150 mg, 0.50 mmol, 1.0 equiv.) in degassed anhydrous C_6_H_6_ (50 mL) under an atmosphere of argon was added AIBN (8.2 mg, 10 mol%). The mixture was heated at reflux for 5 mins and then a solution of *n*Bu_3_SnH (0.20 mL, 0.75 mmol, 1.5 equiv.) in degassed anhydrous C_6_H_6_ (1.8 mL) and a solution of AIBN (20.2 mg, 25 mol%) in degassed anhydrous C_6_H_6_ (2.0 mL) were added separately and simultaneously over 16 h (using a syringe pump) to the refluxing solution. The solvent was removed in vacuo and the crude product was purified by column chromatography on anhydrous K_2_CO_3_/silica gel (10% (*v*/*v*)) using 10% Et_2_O/Hexane (*v*/*v*)) to give the title compound as a colourless oil (34.5 mg, 0.16 mmol, 31%). **R_f_** = 0.31 (10% Et_2_O/Hexane (*v*/*v*)). **δ_H_** (300 MHz, CDCl_3_): 7.68–7.24 (m, 5H, Ar-H), 3.32–3.29 (m, 1H), 2.99–2.96 (m, 1H), 2.59–2.53 (m, 2H), 2.52–2.48 (m, 1H), 2.46–2.42 (m, 1H), 2.40–2.36 (m, 1H), 1.63–1.58 (m, 2H), 1.53–1.49 (m, 2H). **δ_C_** (75 MHz, CDCl_3_): 208.1 (C=O), 141.7 (q, Ar-C), 128.6, 128.3, 126.0 (Ar-C), 47.4 (CH_2_), 39.4 (CH), 38.1, 35.9, 33.7, 29.9 (CH_2_). *v*_max_ (ATR)/cm^−1^: 1163 (C-S), 1702 (C=O). *m*/*z* **HRMS** (ESI^+^) calcd for C_13_H_16_OS = 220.0922 ([M])^+^. Found 220.0920. 

*(E)-S-(5-Phenylpent-2-en-1-yl) 2-bromopropanethioate* (**11**): To EDC (310 µL, 2.0 mmol, 2.0 equiv.) in anhydrous THF (5.5 mL), under argon was added MeBrAcOH (306 mg, 2.0 mmol, 2.0 equiv.). The mixture was stirred for 15 min and then **6** (178 mg, 1.0 mmol, 1.0 equiv.) and DMAP (61 mg, 0.50 mmol, 0.5 equiv.) in anhydrous THF (5.5 mL) under an atmosphere of argon were added. The mixture was stirred at rt for 14 h, then diluted with Et_2_O (150 mL), filtered through a plug of silica and the solvent removed in vacuo. The crude product was purified by column chromatography on silica gel using 4% EtOAc/Hexane (*v*/*v*) to furnish **11** (253 mg, 0.81 mmol, 81%) as a colourless oil. **R_f_** = 0.43 (10% EtOAc/Hexane). **δ_H_** (400 MHz, CDCl_3_): 7.33–7.25 (m, 2H, Ar-H), 7.23–7.15 (m, 3H, Ar-H), 5.80–5.70 (m, 1H, =CH-), 5.52–5.41 (m, 1H, =CH-), 4.51 (q, *J* = 6.9 Hz, 1H, -CHBr), 3.55 (d, *J* = 7.1 Hz, 2H, -CH_2_S), 2.69 (t, *J* = 7.7 Hz, -CH_2_Ph), 2.35 (app-q, 2H, -CH_2_CH=), 1.85 (d, *J* = 6.9 Hz, 3H, -CH_3_). **δ_C_** (100 MHz, CDCl_3_): 196.0 (C=O), 141.6 (q, Ar-C), 134.5 (=CH-), 128.5, 128.4, 125.9 (Ar-C), 124.3 (=CH-), 48.1 (-CHBr), 35.5 (-CH_2_Ph), 34.1 (-CH_2_CH=) 32.1 (-CH_2_S), 22.1 (-CH_3_). **ν_max_** (ATR)/cm^−1^: 641 (C-Br), 1096 (C-S), 1690 (C=O). *m*/*z* **HRMS** (APCI+) calcd for C_14_H_18_OSBr = 313.0257 [(M + H)]^+^. Found 313.0256.

*(E)-S-(5-Phenylpent-2-en-1-yl) propanethioate* (**12**): To a stirred solution of **11** (156 mg, 0.50 mmol, 1.0 equiv.) in degassed anhydrous C_6_H_6_ (50 mL) under an atmosphere of argon was added AIBN (8.2 mg, 10 mol%). The mixture was heated at reflux for 5 min and then a solution of *n*Bu_3_SnH (0.20 mL, 0.75 mmol, 1.5 equiv.) in degassed anhydrous C_6_H_6_ (1.8 mL) and a solution of AIBN (20.2 mg, 25 mol %) in degassed anhydrous C_6_H_6_ (2.0 mL) were added separately and simultaneously over 16 h (using a syringe pump) to the refluxing solution. The solvent was removed in vacuo and the crude product was purified by column chromatography on anhydrous K_2_CO_3_/silica gel (10% (*v*/*v*)) using 10% Et_2_O/Hexane (*v*/*v*)) to give the title compound as a colourless oil (106 mg, 0.455 mmol, 91%). **R_f_** = 0.42 (10% Et_2_O/Hexane (*v*/*v*)). **δ_H_** (400 MHz, CDCl_3_): 7.33–7.25 (m, 2H, Ar-H), 7.23-7.25 (m, 3H, Ar-H), 5.76–5.67 (m, 1H, =CH^−^), 5.52–5.42 (m, 1H, =CH-), 3.51 (d, *J* = 7.1 Hz, 2H, -CH_2_S), 2.69 (t, *J* = 7.7 Hz, 2H, -CH_2_Ph), 2.58 (q, *J* = 7.5 Hz, 2H, -CH_2_C=O), 2.34 (app-q, 2H, -CH_2_CH=), 1.98 (t, *J* = 7.5 Hz, 3H, -CH_3_). **δ_C_** (100 MHz, CDCl_3_): 199.8 (C=O), 141.7 (q, Ar-C), 133.5 (=CH-), 128.5, 128.3, 125.9 (Ar-C), 125.4 (=CH-), 37.3 (-CH_2_C=O), 35.6 (-CH_2_Ph), 34.1 (-CH_2_CH=), 31.0 (-CH_2_S), 9.7 (-CH_3_). ***v*_max_**(ATR)/cm^−1^: 1692 (C=O). *m*/*z* **HRMS** (APCI^+^) calcd for C_14_H_19_OS = 235.1152 ([M + H])^+^. Found 235.1151.

*(E)-S-(5-phenylpent-2-en-1-yl) 2-((ethoxycarbonothioyl)thio)ethanethioate* (**13**): To a stirred solution of **6** (298 mg, 1.0 mmol, 1.0 equiv.) in EtOH (10 mL) at 0 °C was added KSCSOEt (160 mg, 1.0 mmol, 1.0 equiv), and the reaction was stirred at this temperature for 1.5 h. The mixture was then filtered, and the solvent removed in vacuo to yield **13** as a colourless wax (320 mg, 0.94 mmol, 94%) without the requirement for further purification. **δ_H_** (400 MHz, CDCl_3_): 7.34–7.26 (m, 2H, Ar-H), 7.24–7.16 (m, 3H, Ar-H), 5.79–5.70 (m, 1H, =CH-), 5.51–5.42 (m, 1H, =CH-), 4.68 (q, *J* = 7.1 Hz, 2H, -CH_2_O), 4.13 (s, 2H, -CH_2_C=O), 3.56 (d, *J* = 7.1 Hz, 2H, -CH_2_S), 2.69 (t, *J* = 7.5 Hz, 2H, -CH_2_Ph), 2.35 (app-q, 2H, CH_2_CH=), 1.45 (t, *J* = 7.1 Hz, 3H, CH_3_). **δ_C_** (100 MHz, CDCl_3_): 211.8 (C=S), 193.7 (C=O), 141.6 (q, Ar-C), 134.3 (=CH-), 128.4, 128.3, 125.9 (Ar-C), 124.5 (=CH-), 70.9 (OCH_2_-), 45.6 (-CH_2_C=O), 35.5 (-CH_2_Ph), 34.0 (-CH_2_CH=), 32.0 (-CH_2_S),13.7 (-CH_3_). *v*_max_ (ATR)/cm^−1^: 1050 (C=S), 1683 (C=O). *m*/*z* **LRMS** ESI^−^: calcd. C_16_H_19_O_2_S_3_ ([M − H]^−^): 339.1. Found 339.1.

## 4. Conclusions

In conclusion, Ueno-Stork cyclisation of α-halo thioacetals cannot be achieved, likely due to the radical fragmentation that occurs upon formation of the aluminium thioacetal. Evidence supporting this theory can be derived from the fragmentation/rearrangement of selanyl compound **9**. However, a method for the radical cyclisation of α-bromo-allylic thioesters has been reported, though this produces thiolactones in poor yield. Additionally, α substitution does not appear to be tolerated and the reaction does not appear to be general. Work is currently ongoing for the optimisation of this cyclisation process and the xanthate group cyclisation strategy. Furthermore, alternative cyclisation routes, such as atom-transfer-cyclisation methods are also being pursued. 

## Supplementary Materials

The following are available online, ^1^H- and ^13^C-NMR Spectra for Compounds **5**, **6**, **7**, **9**, **11**, **12** and **13**.
