# Supplementary material for: Radical-Mediated Reactions of α-Bromo Aluminium Thioacetals, α-Bromothioesters, and Xanthates for Thiolactone Synthesis
Source: Molecules. 2018 Apr 13;23(4):897. doi: 10.3390/molecules23040897 (PMC6017948; doi:10.3390/molecules23040897)
Supplement: Supplementary file 1 [file molecules-23-00897-s001.pdf]

SUPPLEMENTARY MATERIAL for

Radical Mediated Rearrangement of α-Bromo Aluminium Thioacetals

Ruairí O. Mc Court, Fabrice Dénès, and Eoin M. Scanlan *

Index

Figures S1-S15 ^{1}H and ^{13}C-NMR Spectra for Compounds 5, 6, 7, 9, 11, 12 and 13 1

Figures S1-S15 1H and 13C-NMR Spectra for Compounds 5, 6, 7, 9, 11, 12 and 13

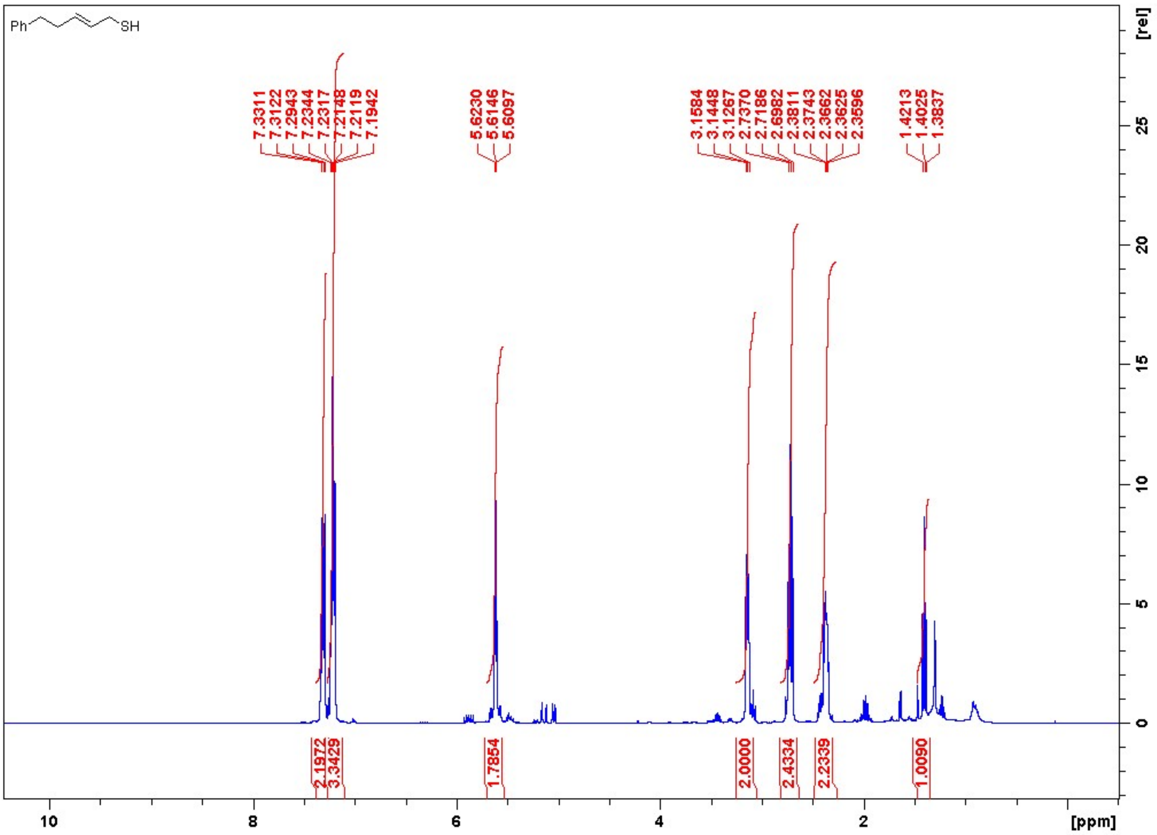

Figure S1. ^{1}H NMR (400 MHz) spectrum in $CDCl_3$ of **5**.

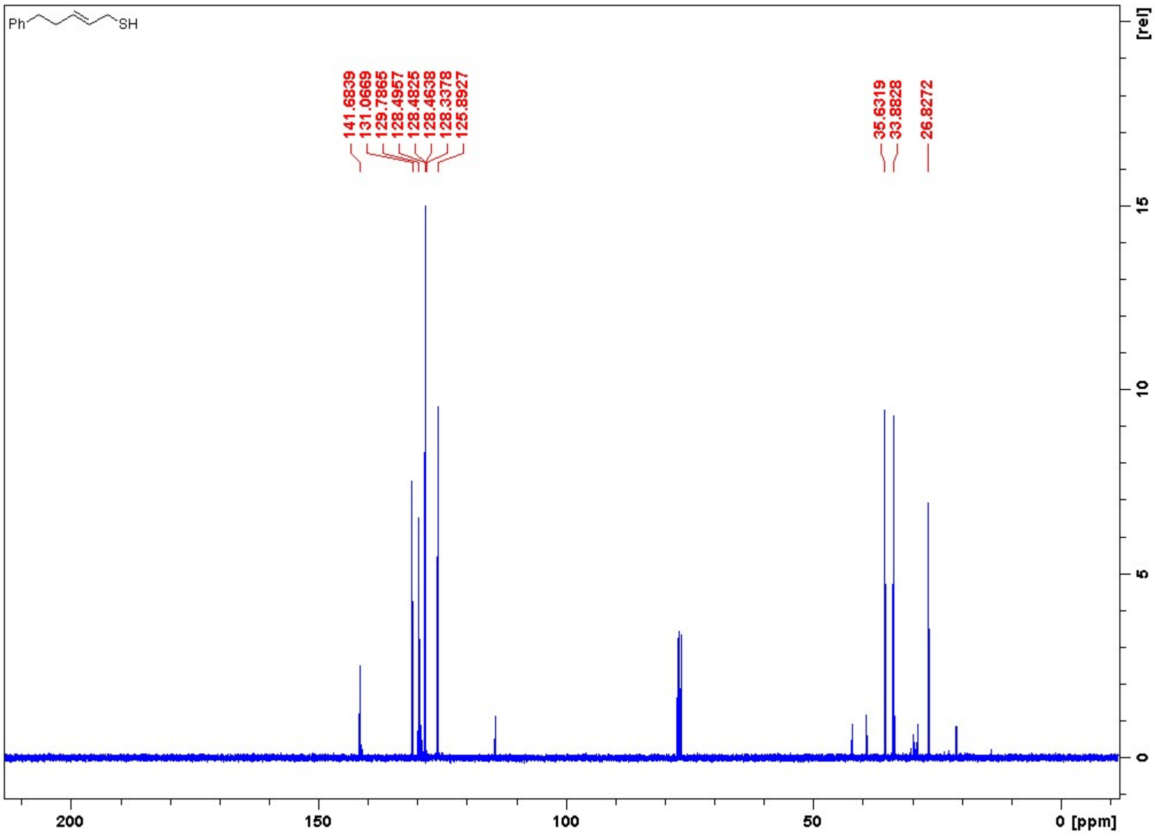

Figure S2. ^{13}C NMR (101 MHz) spectrum in $CDCl_3$ of **5**.

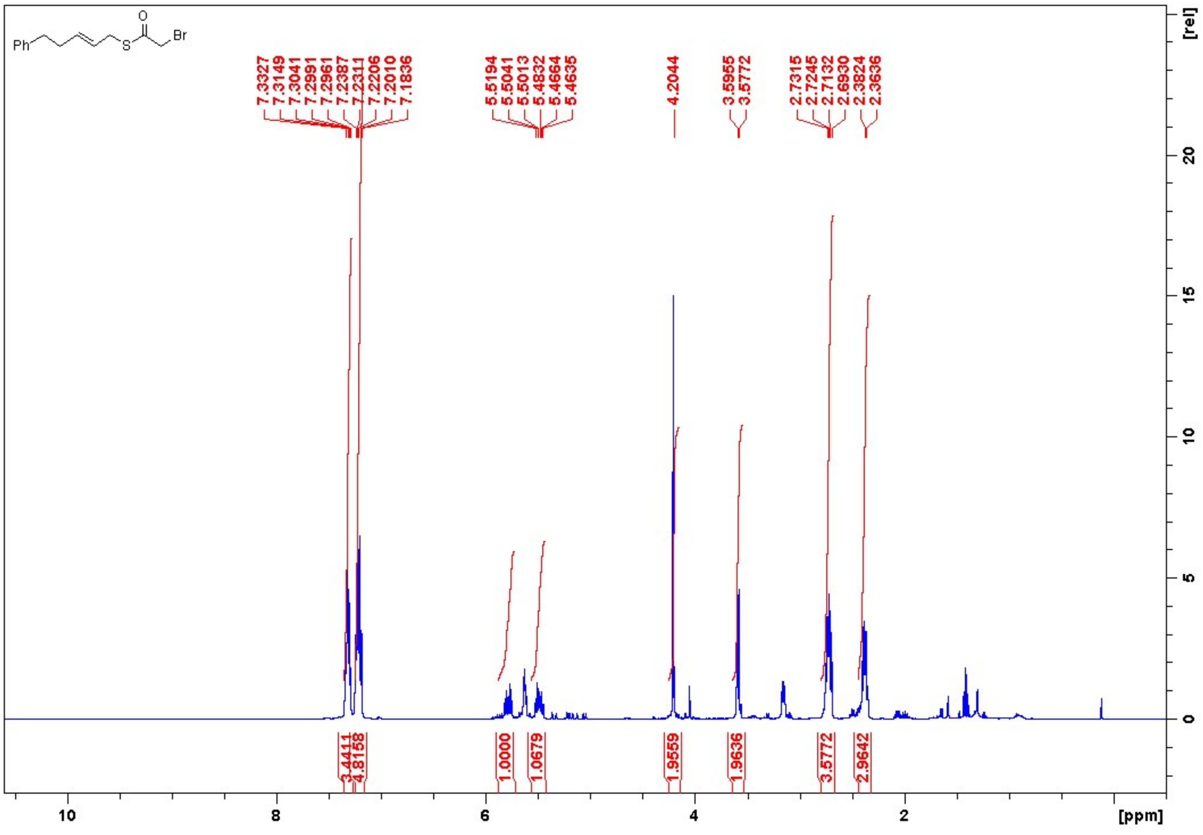

Figure S3. ^{1}H NMR (400 MHz) spectrum in $CDCl_3$ of **6**.

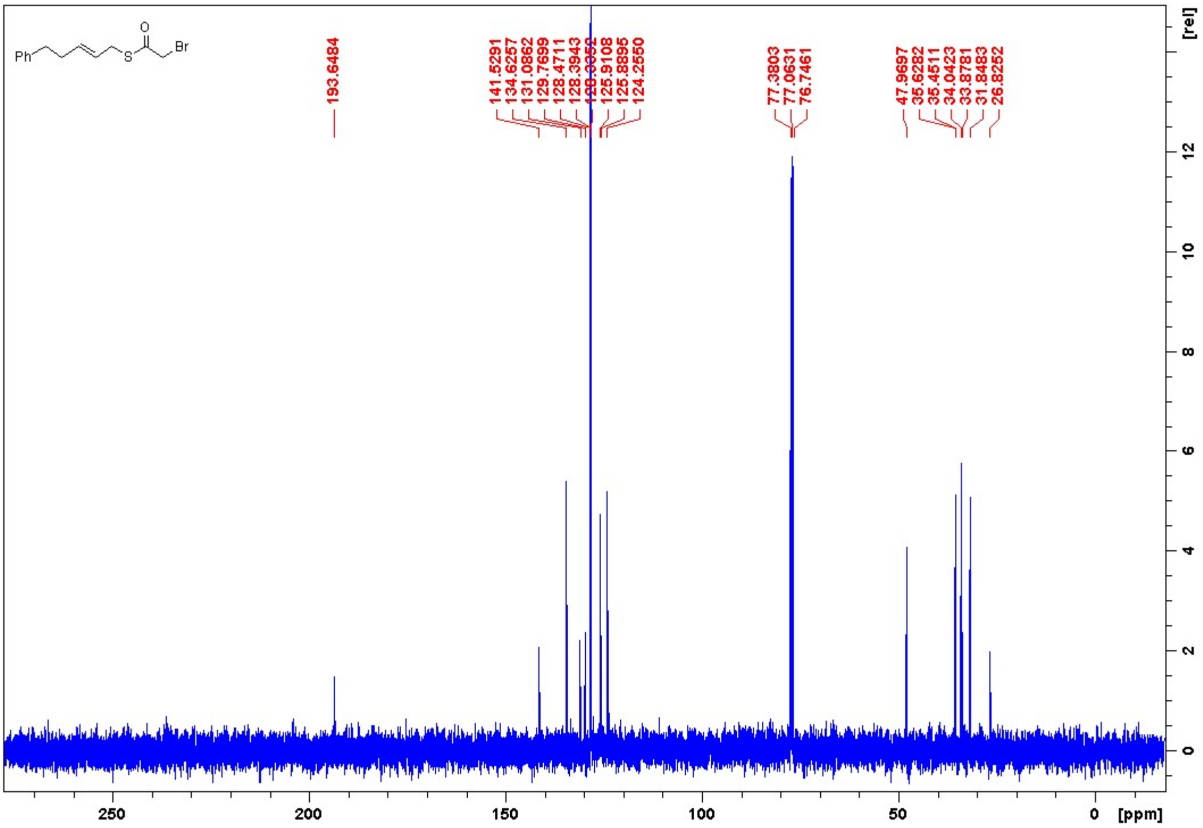

Figure S4. ^{13}C NMR (101 MHz) spectrum in $CDCl_3$ of **6**.

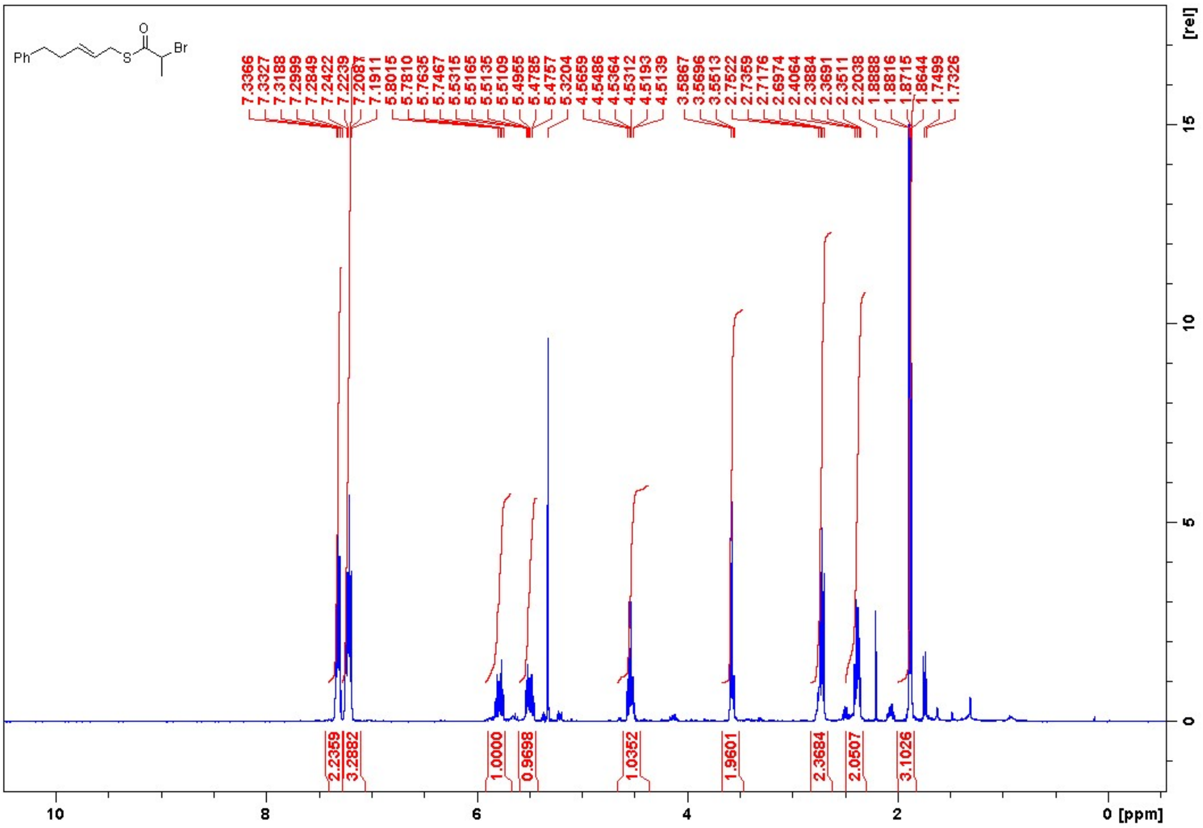

Figure S5. ^{1}H NMR (400 MHz) spectrum in $CDCl_3$ of **11**.

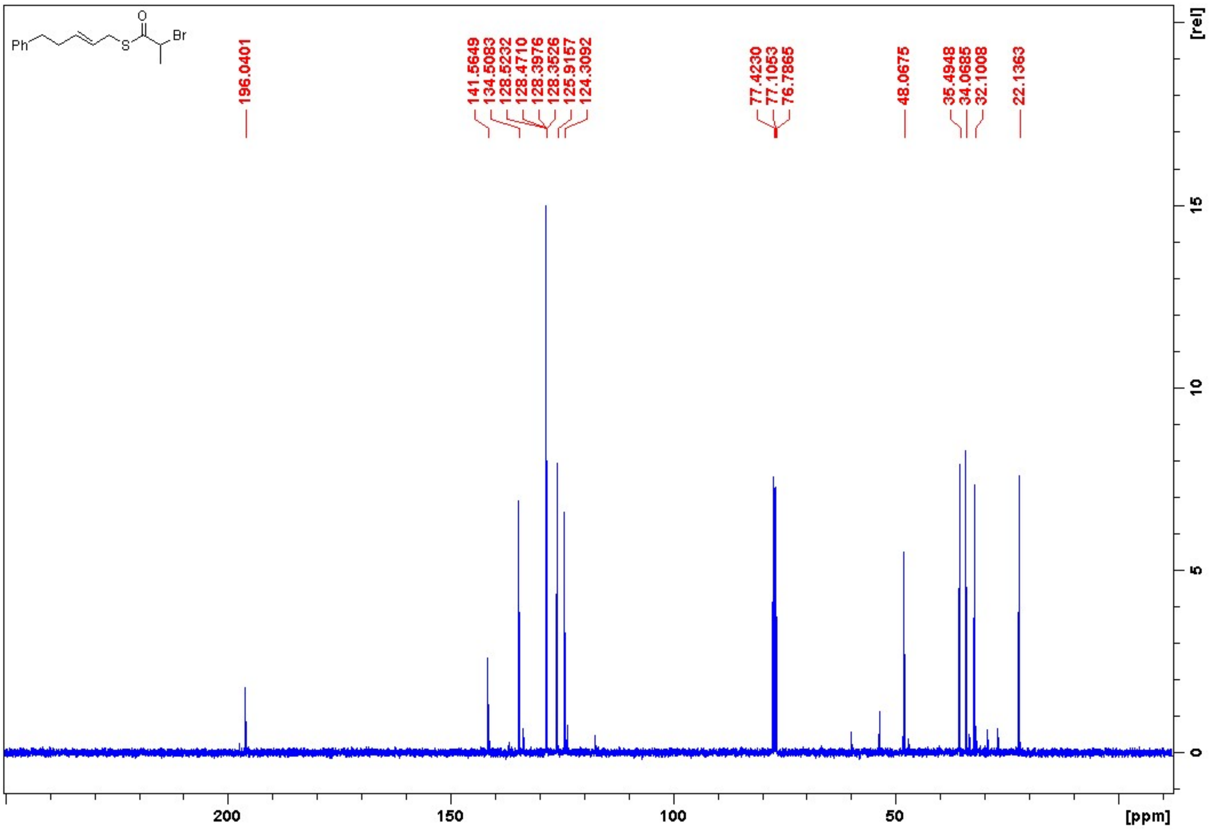

Figure S6. ^{13}C NMR (101 MHz) spectrum in $CDCl_3$ of **11**.

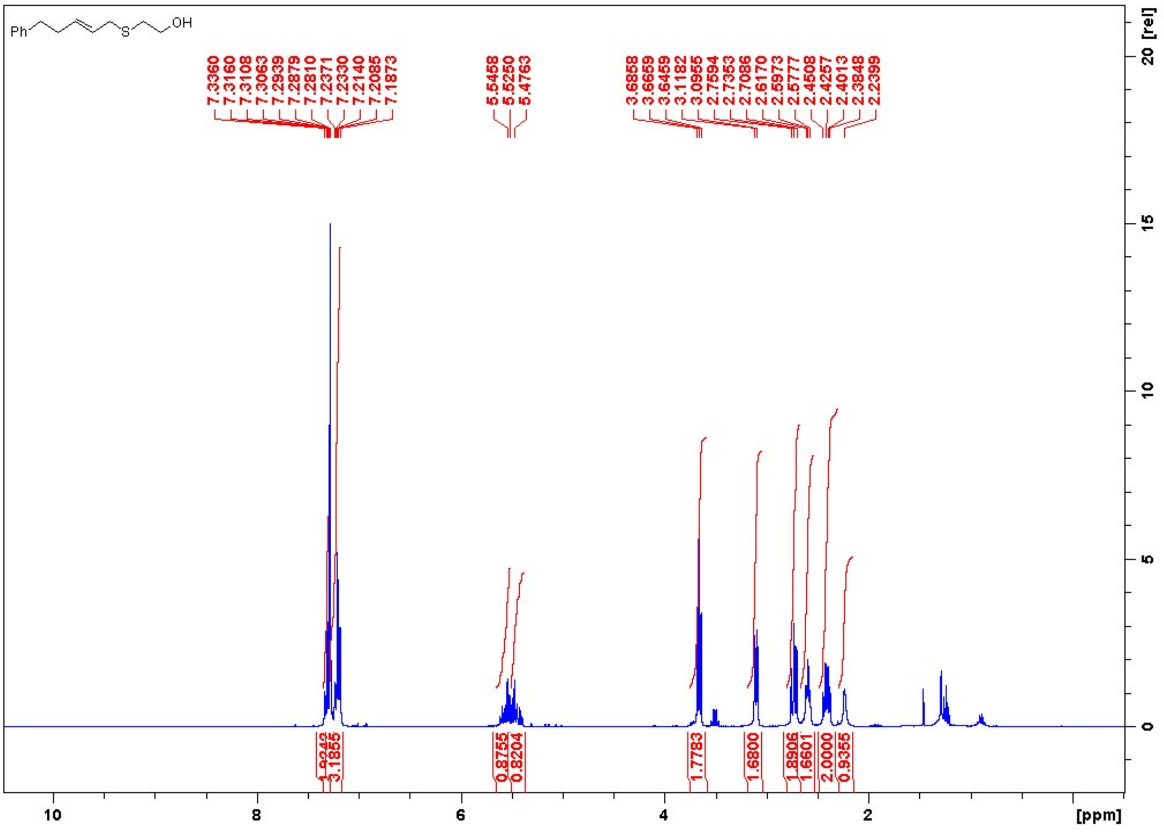

Figure S7. ^{1}H NMR (400 MHz) spectrum in $CDCl_3$ of **7**.

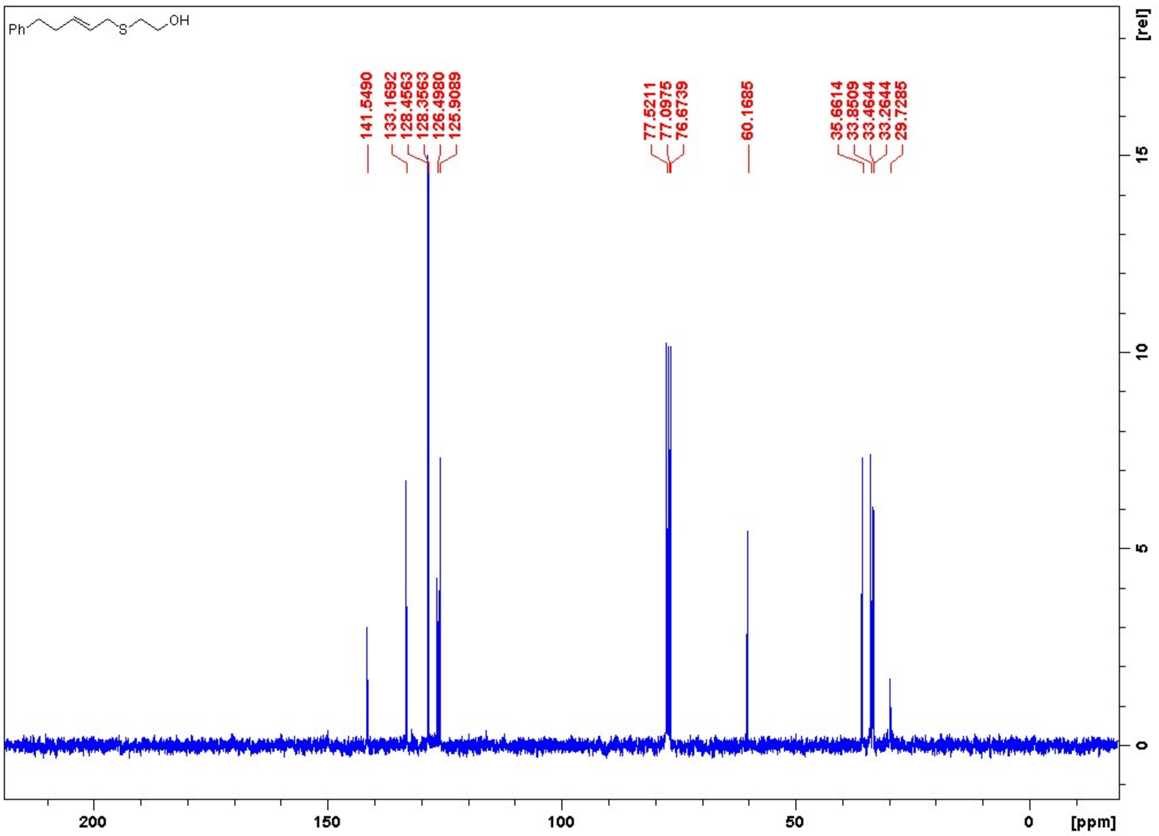

Figure S8. ^{13}C NMR (101 MHz) spectrum in $CDCl_3$ of **7**.

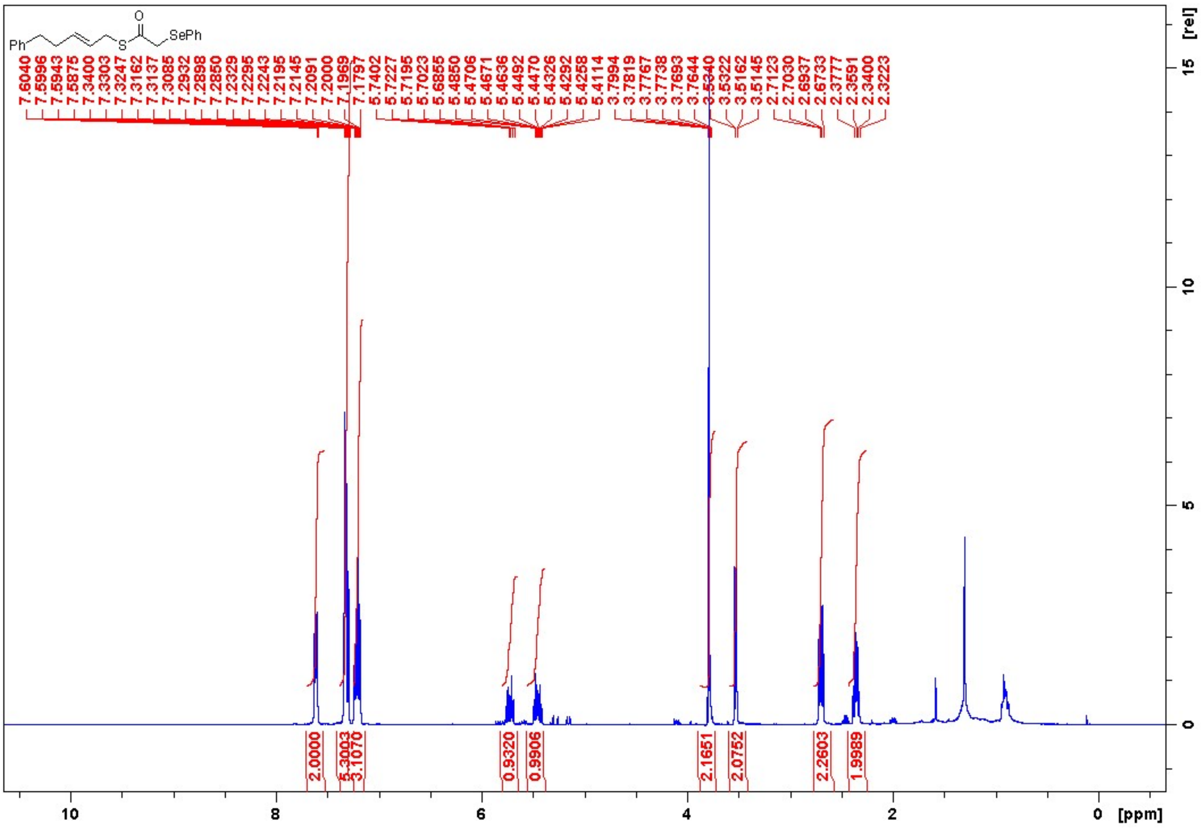

Figure S9. ^{1}H NMR (400 MHz) spectrum in $CDCl_3$ of **9**.

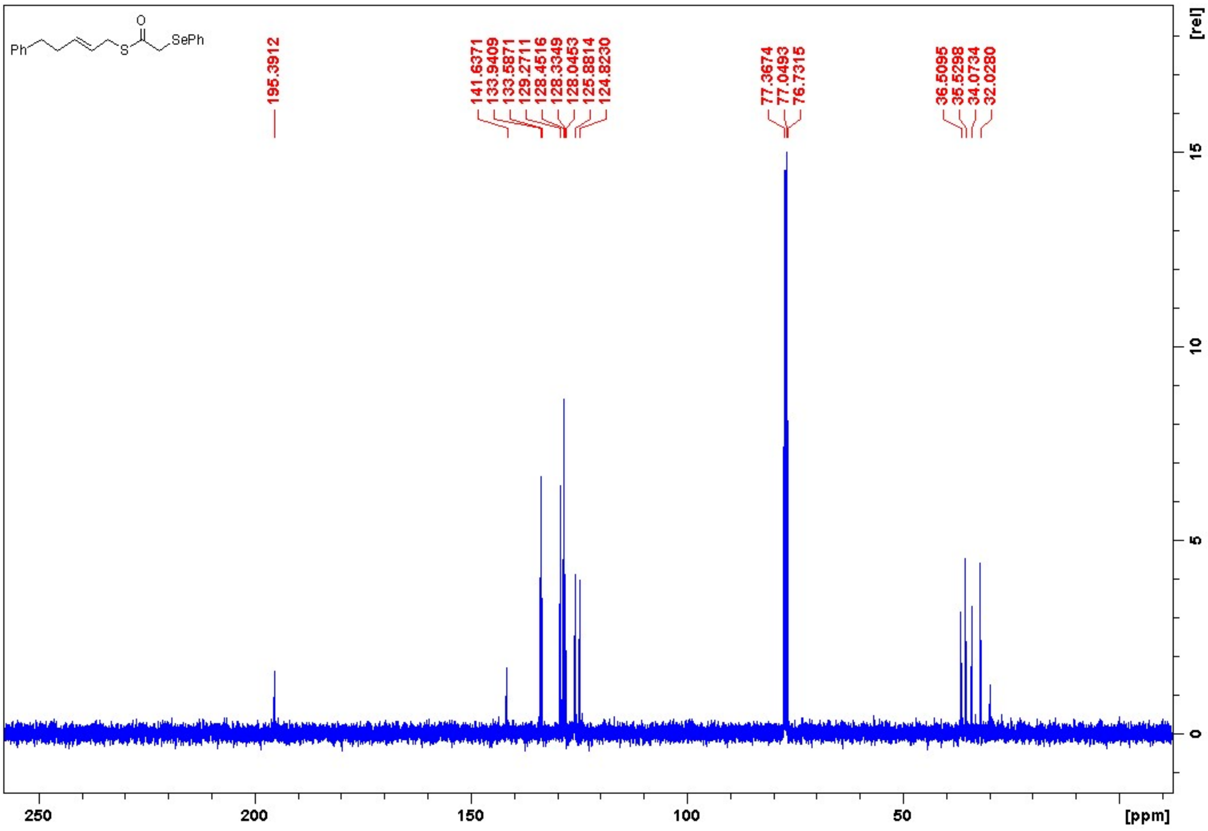

Figure S9. ^{13}C NMR (101 MHz) spectrum in $CDCl_3$ of **9**.

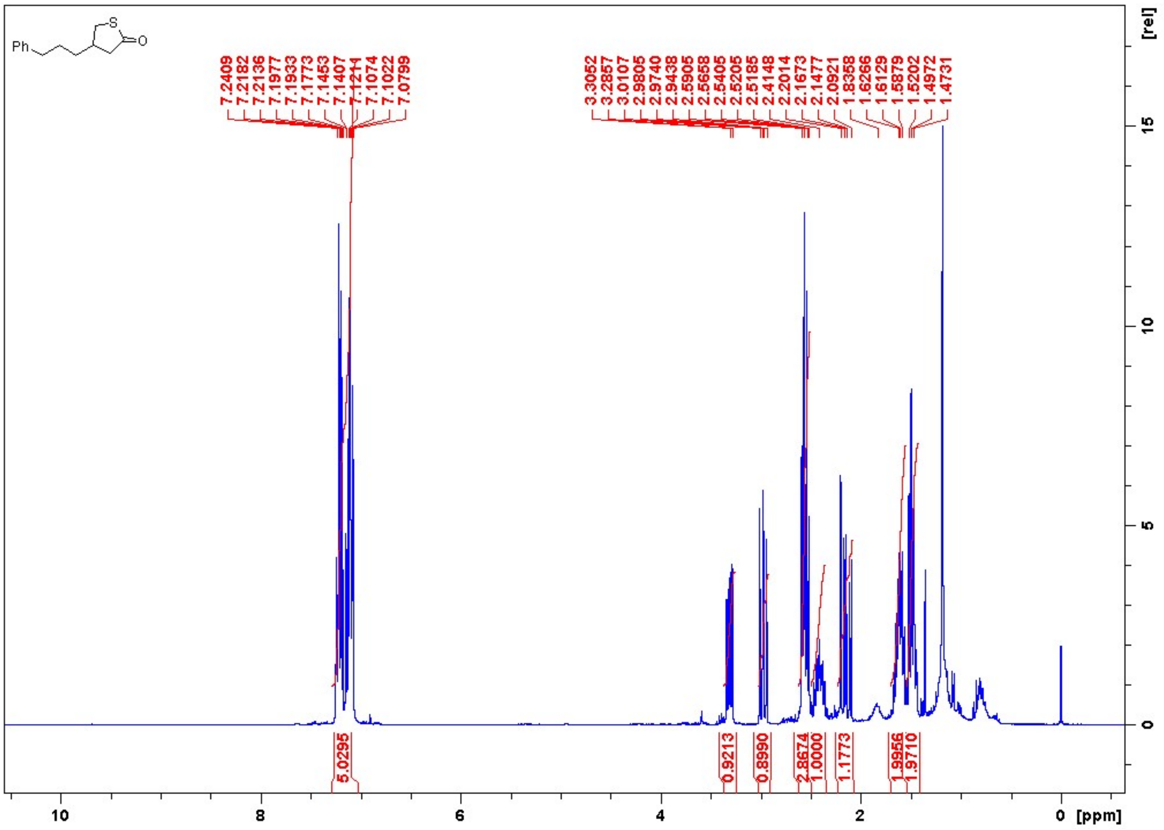

Figure S10. ^{1}H NMR (400 MHz) spectrum in $CDCl_3$ of **10**.

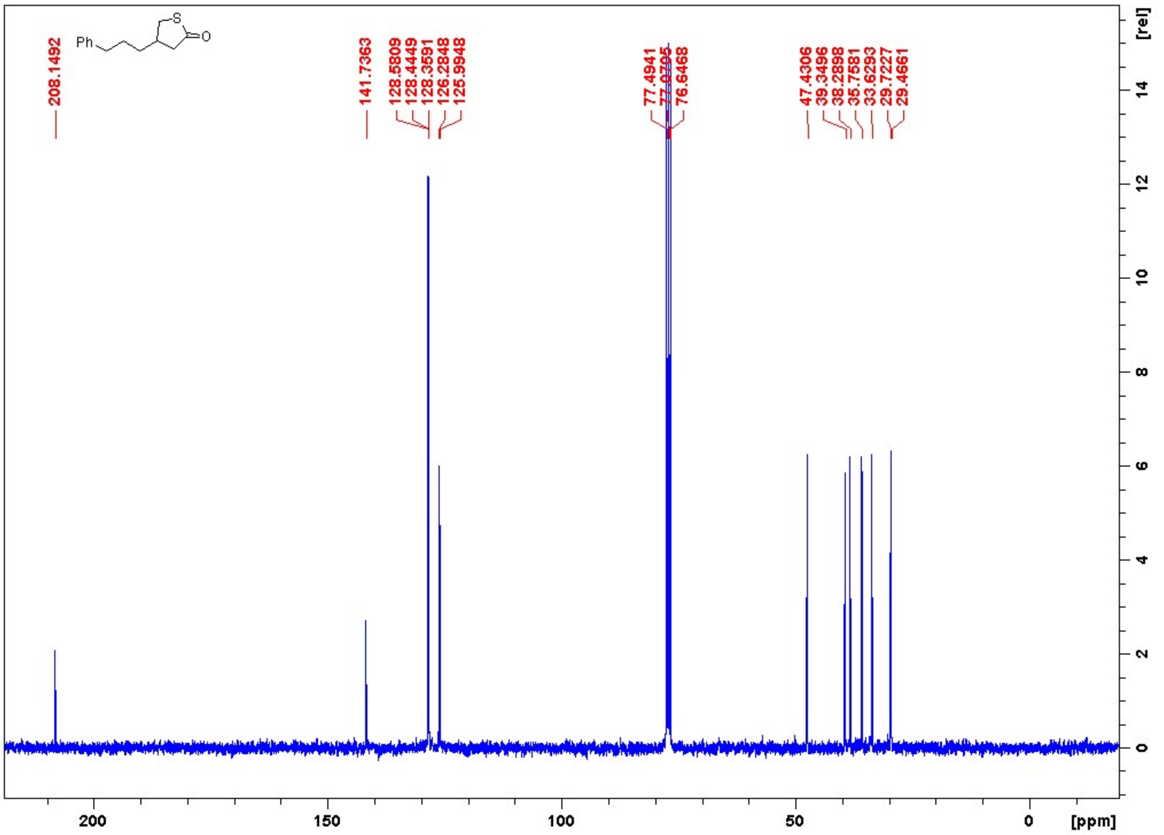

Figure 11. ^{13}C NMR (101 MHz) spectrum in $CDCl_3$ of **10**.

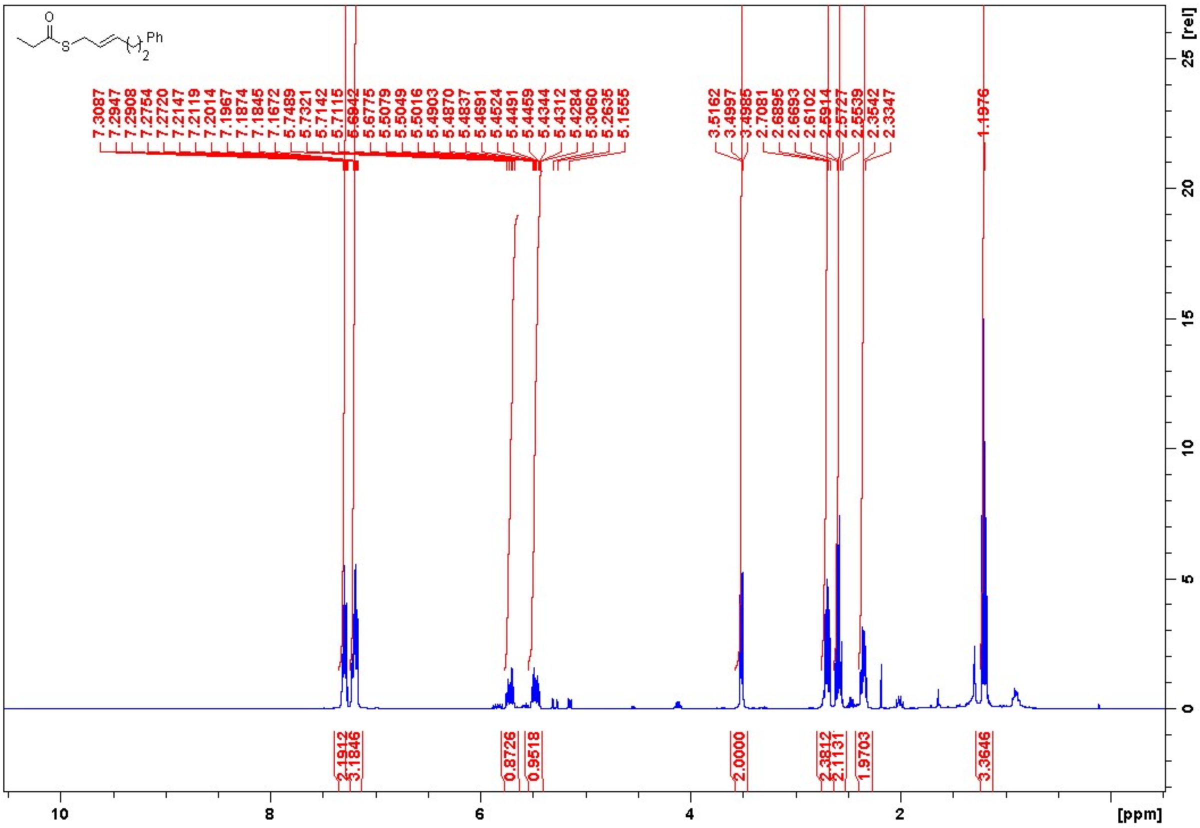

Figure S12. ^{1}H NMR (400 MHz) spectrum in $CDCl_3$ of **12**.

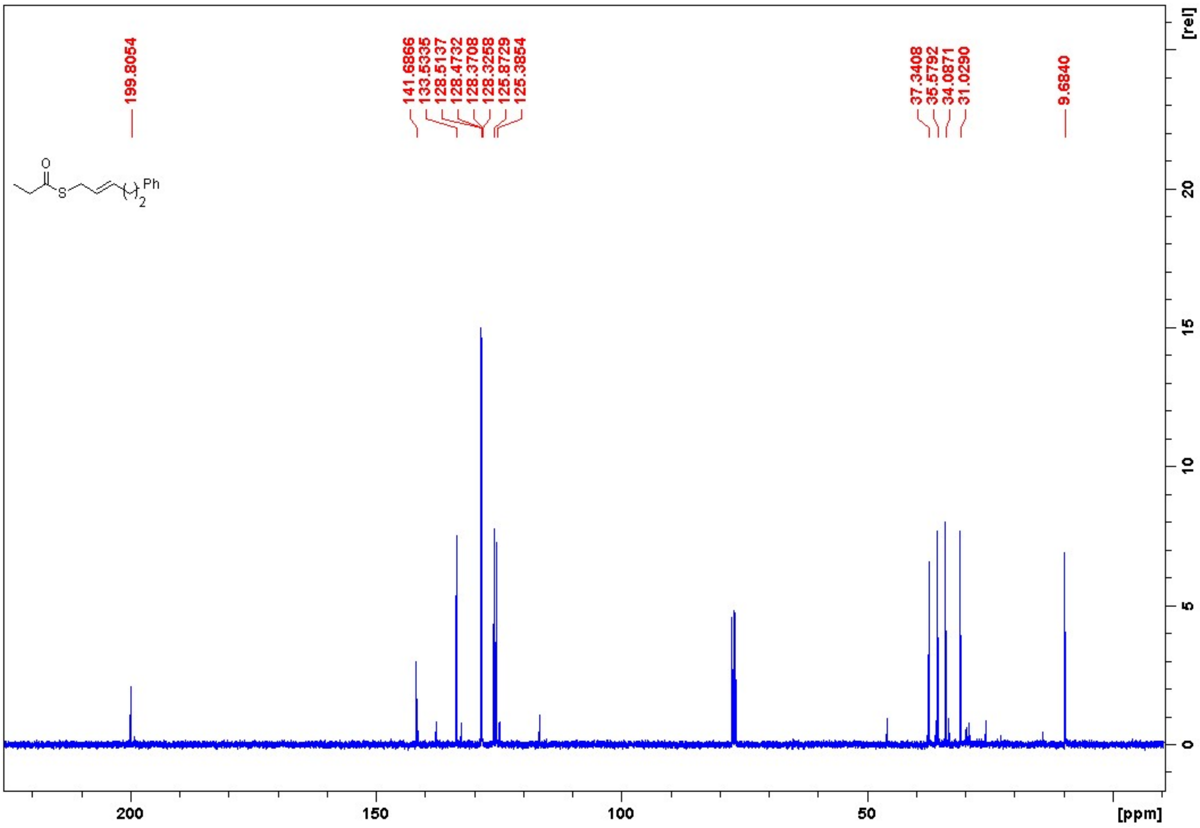

Figure 13. ^{13}C NMR (101 MHz) spectrum in $CDCl_3$ of **12**.

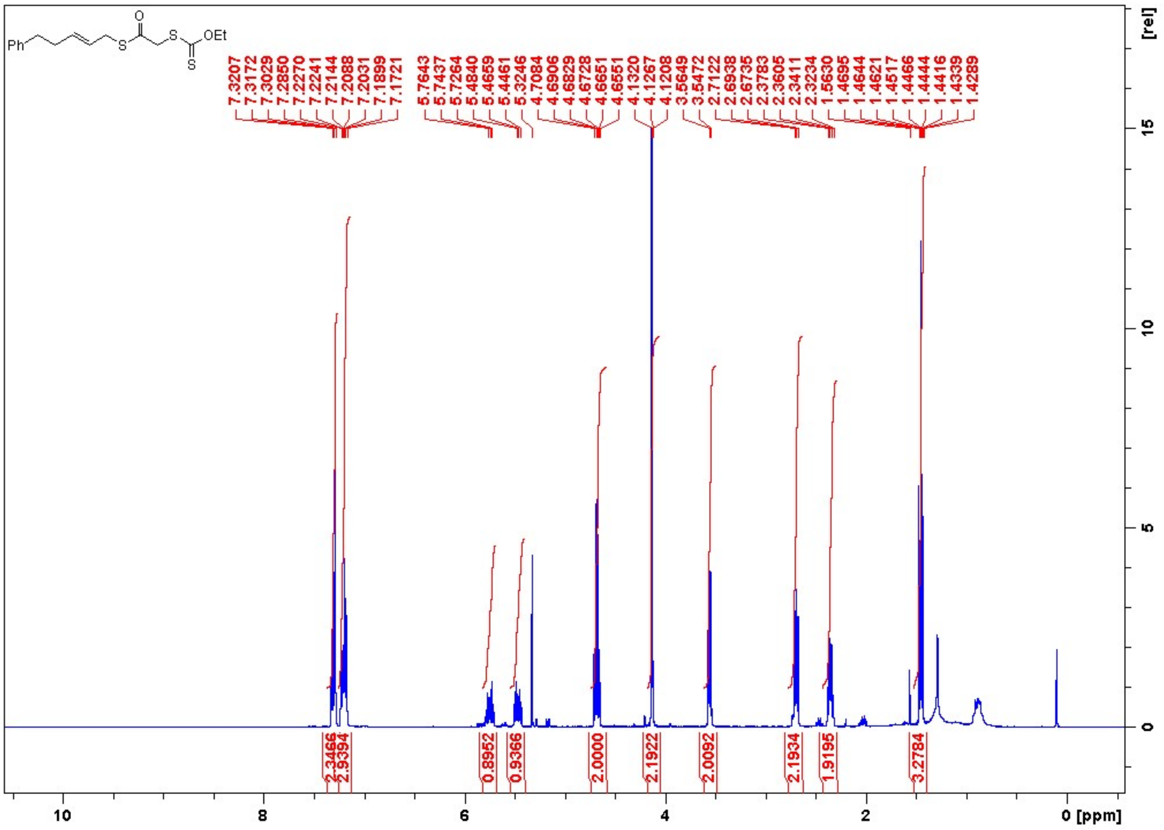

Figure S14. ^{1}H NMR (400 MHz) spectrum in $CDCl_3$ of **13**.

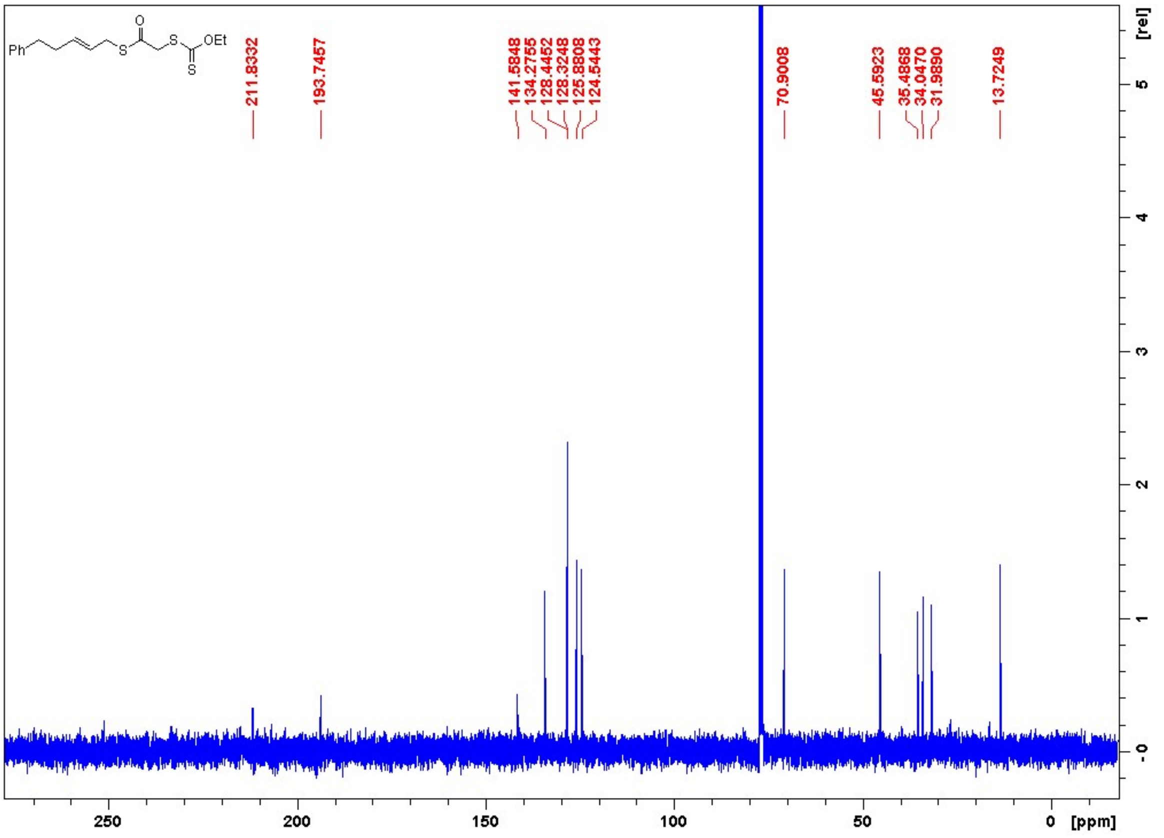

Figure 15. ^{13}C NMR (101 MHz) spectrum in $CDCl_3$ of **13**.
